# Root functional traits dominate maize seedling growth responses to different humic acids

**DOI:** 10.3389/fpls.2026.1908981

**Published:** 2026-08-20

**Authors:** Zhaolong Ding, Ruijia Guo, Aimaieraili Tuheti, Huawei Shao, Chunhui Ge, Jifeng Zhang

**Affiliations:** 1 Institute of Agricultural Resources and Environment, Xinjiang Academy of Agricultural Sciences, Urumqi, Xinjiang, China; 2 Aksu Regional Agricultural Technology Extension Center of Xinjiang Uygur Autonomous Region, Aksu, Xinjiang, China

**Keywords:** biomass, humic acid, maize, nutrient accumulation, root functional traits

## Abstract

**Introduction:**

Humic acids (HAs) are natural organic macromolecules that play a critical role in sustainable agricultural production.

**Methods:**

This study compared the effects of five HA productstwo lignin-based biological HAs (acid-modified, LHAac; alkali-modified, LHAal), one mineral-derived HA (MHA), one sludge-derived HA (SHA), and one commercial HA (CHA)on maize seedling growth.

**Results:**

All HA treatments significantly improved root growth, photosynthetic performance, nutrient accumulation, and biomass production. Among them, MHA exhibited the strongest efficacy in promoting root proliferation and increasing specific root length, whereas SHA significantly enhanced root diameter, tissue density, and root activity. HA treatments elevated the net photosynthetic rate (Pn), relative chlorophyll content (SPAD value), and stomatal conductance (Gs) of maize seedlings, while reducing intercellular CO₂ concentration (Ci) in leaves. Mantel correlation analysis and variance partitioning analysis (VPA) revealed that root functional traits accounted for the largest proportion (26.7%) of the variation in seedling biomass, followed by root nutrient status (12.3%). Significant synergistic effects were also observed between photosynthetic parameters and shoot nutrient status, as well as between root-related traits and aboveground physiological indicators.

**Discussion:**

Collectively, HAs from diverse sources improved maize seedling growth through coordinated changes in root morphology, photosynthetic activity, and nutrient utilization; root functional traits made the largest independent contribution to explaining seedling biomass variation among all measured indicators.

## 1 Introduction

Balancing high crop productivity with the maintenance of soil health has become a major challenge in modern agriculture (Zhang et al., 2013; Lal, 2015). The long-term dependence on chemical fertilizers and intensive cultivation has led to soil degradation, nutrient imbalance, and declining water-use efficiency, posing serious threats to agricultural sustainability, and it is urgent to explore sustainable agricultural management measures (FAO, 2011). In recent years, organic soil conditioners (especially humic acids (HAs)) have gradually garnered extensive attention in agricultural research and practice due to their significant soil improvement effects and eco-friendliness (Jarosz et al., 2025; Zhao et al., 2025). As global agriculture shifts toward resource-efficient and eco-safe production systems, the exploration of HAs as multifunctional soil conditioners and plant growth stimulants has become particularly relevant.

HAs are complex organic macromolecules derived from the decomposition and humification of plant and animal residues. Their rich carboxyl and phenolic hydroxyl groups confer strong cation-exchange capacity and redox activity (Stevenson, 1994; Lamar et al., 2024). These chemical features enable HAs to buffer soil pH, enhance nutrient availability, and improve soil aggregation (Lamar et al., 2024; Hao et al., 2025; Sheteiwy et al., 2025). Beyond soil improvement, HAs directly influence plant metabolism by modulating root morphology, ion transport, and hormonal signaling (Juan et al., 2024; Zhou et al., 2025). Acting in a hormone-like manner, HAs stimulate root elongation and lateral branching by activating plasma membrane H^+^-ATPase and auxin-related pathways, thereby expanding root surface area and improving nutrient uptake efficiency (Zandonadi et al., 2025). The anionic functional groups of HAs can also form complexes with nitrogen, phosphorus, and potassium ions, preventing nutrient fixation and increasing their bioavailability (Wang et al., 2017; Mindari et al., 2024; Nabi et al., 2025). HAs exert adsorption and buffering effects on potassium ions, thereby increasing the supply of available potassium in soil (Guo et al., 2025; Mondher and Oleiwi, 2025). Furthermore, HAs enhance microbial and enzymatic activity in the rhizosphere, accelerating the decomposition and release of organic nutrients for plant use (Jing et al., 2021; Zhang et al., 2024b; Höfle et al., 2024; da Silva et al., 2025). Through these combined effects, HAs have been shown to improve photosynthetic efficiency, increase chlorophyll synthesis, and ultimately enhance plant biomass accumulation (Souza et al., 2022; Rathor et al., 2024; Zandonadi et al., 2025; Zhang et al., 2024a; Ali et al., 2025). However, studies have also reported that HA application does not always increase crop yield and may even exert negative effects under certain conditions (Mahoney et al., 2017; Ma et al., 2024). Such inconsistencies likely arise from variations in HA source materials and extraction methods.

HAs used in agriculture are generally classified into two broad categories: (i) mineral-derived HAs, extracted from lignite, weathered coal, or peat; and (ii) biological HAs, produced from plant residues, animal by-products, or organic wastes through biological fermentation or chemical conversion (MacCarthy, 2001; de Melo et al., 2016; Nardi et al., 2021). In addition, sludge-derived HAs, obtained from wastewater treatment byproducts, have recently attracted attention due to their high nutrient content and functional-group diversity. Studies have shown that both mineral and biological HAs can improve soil fertility and promote crop growth, but their efficacy and mechanisms often differ (Akimbekov et al., 2020; Kumar Sootahar et al., 2020; Tavares et al., 2021). Mineral HAs are typically richer in aromatic rings and condensed structures, enhancing redox capacity and stress tolerance, whereas biological and sludge HAs often possess lower molecular weight and more reactive functional groups, conferring greater bioavailability and hormonal activity (Liang et al., 2021; Nardi et al., 2021; Chen et al., 2022; Cai et al., 2023). These source-dependent compositional differences likely result in distinct physiological responses in plants, particularly in the root system, which serves as the primary interface between soil amendments and plant metabolism.

The positive effects of HA in agricultural production have been fully demonstrated. However, most studies have focused on the effects of HA from a single source, with few systematic comparisons across HA of different origins. A systematic and comprehensive comparison of the effects of HAs from different sources on crop growth, as well as the underlying mechanisms, is of great significance for optimizing the development and application of HA products. Therefore, five HA products (two lignin-derived biological HAs, one mineral-derived HA, one sludge-derived HA, and one commercial HA) were selected for a pot experiment. The objectives were to: (1) elucidate the differential effects of HAs from diverse sources on maize seedling growth; (2) identify the key factors driving these differences.

## 2 Materials and methods

### 2.1 Experimental materials

The soil used was a gray desert soil, collected from the Gray Desert Soil experimental base of the Xinjiang Uygur Autonomous Region Academy of Agricultural Sciences. The sampled soil was cleared of stones and plant residues, thoroughly mixed, air-dried, and sieved through a 2 mm mesh. The basic physicochemical properties of the soil were: organic matter 18.6 g kg-1, alkali-hydrolyzable nitrogen 47.53 mg kg-1, available phosphorus 28 mg kg-1, and available potassium 201 mg kg-1. Five humic acid (HA) materials with different sources and preparation methods were used in this study, and their detailed preparation procedures are presented below: (1) Mineral-derived HA (MHA, 50% HA content) was prepared from weathered coal. Following hydrogen peroxide oxidation pretreatment, the raw material was purified via alkali dissolution and acid precipitation to obtain small molecule-enriched MHA. (2) Sewage sludge-derived HA (SHA, 32% HA content) was synthesized through a hydrothermal reaction of sewage sludge with alkaline reagents under high temperature and pressure. The humic fractions were released by alkali dissolution, and SHA was finally harvested after solid-liquid separation and acidification precipitation. (3) Acid-modified lignin-based HA (LHAac, 25% HA content) was fabricated via acidic modification and degradation of lignin using organic acid and sodium metabisulfite, followed by purification and concentration. (4) Alkali-modified lignin-based HA (LHAal, 25% HA content) was obtained by oxidative modification of lignin in a potassium hydroxide-sodium percarbonate alkaline system. The organic structure of lignin was degraded and reconstructed, and purified LHAal was collected subsequently. (5) Commercial HA (CHA, 50% HA content) was purchased from Xinjiang Xinlianxin Energy Chemical Co., Ltd.

### 2.2 Experimental design and implementation

Six treatments with three replicates each were established in this experiment: (1) CK (control, no HA addition); (2) LHAac, acid-modified lignin-based bio-HA; (3) LHAal, alkali-modified lignin-based bio-HA; (4) MHA, mineral-derived HA; (5) SHA, sewage sludge-derived HA; (6) CHA, commercial HA product. Polyethylene pots (23 cm diameter, 21.5 cm height) were used for the pot trial, and each pot was filled with an equal mass of test soil. Uniform amounts of basal fertilizers were incorporated into the soil of all treatments, including analytical-grade urea (CO(NH_2_)_2_) at 150 mg kg⁻¹ and analytical-grade potassium dihydrogen phosphate (KH_2_PO_4_) at 75 mg kg⁻¹. Uniform, plump, and undamaged maize seeds were pre-germinated in a constant-temperature incubator for 2 days, and six germinated seeds were sown evenly in each pot. When seedlings reached the four-leaf stage, three healthy seedlings of consistent size were retained per pot. All HA treatments were applied via root irrigation following the principle of equal HA dosage. LHAac and LHAal solutions were diluted 6000-fold, whereas MHA, SHA and CHA solutions were diluted 15000-fold. Except for the CK treatment, which received an equal volume water, each pot was irrigated with the corresponding HA solution at an identical volume to ensure consistent total HA input across all HA treatments. The experiment was carried out in a glass greenhouse under natural light conditions. The temperature was maintained at 25–30 °C during the day and 18–22 °C at night, with relative humidity ranging from 60% to 70%. Soil water content was adjusted daily by weighing to maintain 70% of field capacity. Pots were randomly repositioned every three days to minimize positional effects. Throughout the experimental period, all management practices were kept identical among treatments.

### 2.3 Sample collection and analysis

Plant samples were harvested at 45 days: Maize is undergoing a critical transition from the seedling stage to the jointing stage at this growth stage, and this time can better reflect the comprehensive effect of HA treatment on seedling growth. Complete maize seedlings were carefully removed from the pots, and soil adhering to roots was thoroughly rinsed with water.

Root activity was determined using the 2,3,5-triphenyltetrazolium chloride (TTC) reduction method. Fresh root samples underwent a dark staining reaction with TTC; the produced triphenyl formazan (TTF) was extracted with ethyl acetate, and absorbance was measured at 485 nm. Root activity was calculated via a standard curve and expressed as the production rate of TTF per gram of fresh root per hour. Root systems were scanned using an Epson V700 scanner, and images were analyzed with WinRHIZO Pro 2012 (Regent Instruments Inc., Canada) to obtain root morphological parameters, including total root length, average root diameter and root volume. Specific root length (SRL) and root tissue density (RTD) were further calculated:


$$\text{Specific root length}\left(SRL\right)=\text{total root length}\left(m\right)/\text{root dry weight}\left(g\right)$$

$$\text{Root tissue density}\left(RTD\right)=\text{root dry weight}\left(g\right)/\text{root volume}\left(cm³\right)$$

The above-ground and root tissues of maize were oven-dried (30 min at 105 °C for enzyme deactivation, followed by drying at 70 °C to constant weight) and weighed to determine dry biomass. Dried plant samples were ground for the measurement of total nitrogen (N), total phosphorus (P) and total potassium (K) concentrations. Total N was determined by the semi-micro Kjeldahl method; total P was measured using the molybdenum-antimony anti-colorimetric method; total K was quantified via flame photometry.

Leaf gas exchange parameters, including net photosynthetic rate (Pn), stomatal conductance (Gs), transpiration rate (Tr), intercellular CO_2_ concentration (Ci), together with SPAD values (SPAD-502 chlorophyll meter), were measured uniformly between 9:00 and 11:30 a.m. For each maize plant, three fully expanded top leaves were selected. Measurements were conducted on intact, disease-free, healthy leaf tissues in the middle portion, avoiding the main leaf veins. When measurements were performed with the LI-6800 portable photosynthesis system (LI-COR Inc., USA), light intensity and CO_2_ concentration inside the leaf chamber were set to fixed values to ensure consistent measurement conditions across all treatments.

### 2.4 Data analysis

Statistical analysis was performed using SPSS 25.0. The Shapiro–Wilk test and Levene’s test were used to verify data normality and homogeneity of variance. One-way ANOVA was conducted to detect differences in maize seedling indices among various HA treatments, followed by LSD multiple comparisons at the 0.05 significance level. Mantel tests were conducted using the “linkET” package in R (version 4.5.0) to assess correlations between maize biomass and various plant traits, and results were visualized with the “ggplot2” package. Variance partitioning analysis (VPA) was performed using the “vegan” package in R to quantify the contributions of different groups of factors to biomass variation, and the results were visualized accordingly. Graphs were produced using Origin 2025 software. Data in the figures are displayed as mean ± standard error(n=9).

## 3 Results

### 3.1 Root functional traits of maize seedlings under different HA treatments

**Figure 1:**
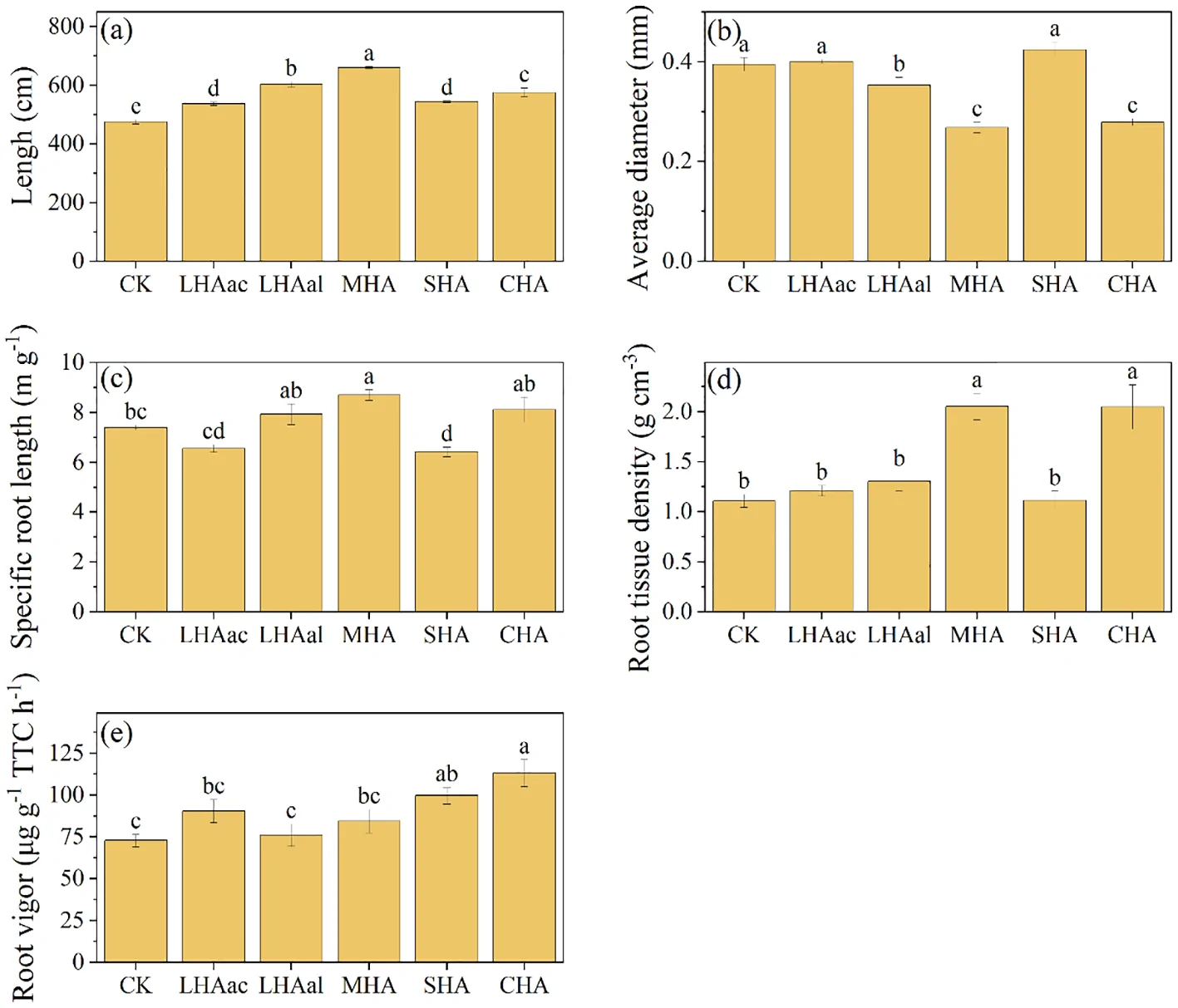
Root functional traits of maize seedlings under different HA treatments. **(a)** Total root length; **(b)** Average root diameter; **(c)** Specific root length; **(d)** Root tissue density; **(e)** Root vigor. Different lowercase letters represent significant differences among treatments.

Compared to the control, HA treatments significantly increased the total root length of maize seedlings by 13.01%–38.80%. The MHA treatment resulted in the greatest total root length (**Figure 1a**). The LHAal, MHA, and CHA treatments significantly reduced the average root diameter of maize seedling roots, whereas SHA resulted in the highest average root diameter (**Figure 1b**). The MHA treatment significantly increased specific root length and had the highest value among all treatments, while the SHA treatment significantly reduced specific root length (**Figure 1c**). The MHA and SHA treatments significantly increased root tissue density (**Figure 1d**). The SHA and CHA treatments also significantly increased root vigor (**Figure 1e**).

### 3.2 Photosynthetic characteristics of maize seedlings under different HA treatments

**Figure 2:**
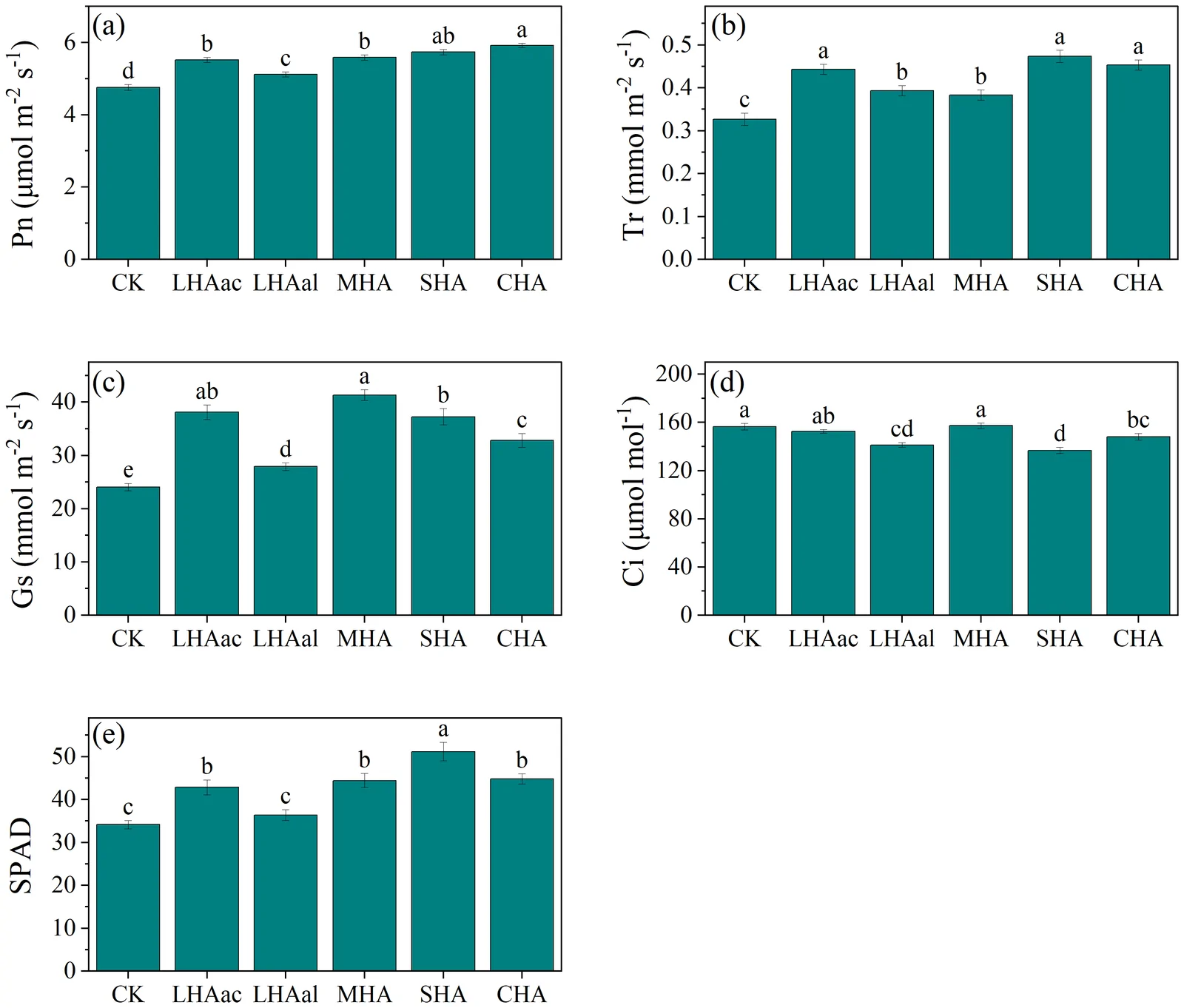
Photosynthetic characteristics of maize seedling leaves under different HA treatments. **(a)** Net photosynthetic rate (Pn); **(b)** Transpiration rate (Tr); **(c)** Stomatal conductance (Gs); **(d)** Intercellular CO_2_ concentration (Ci); **(e)** Relative chlorophyll content (SPAD). Different lowercase letters represent significant differences between treatments.

All HA treatments significantly increased the Pn, Tr, and Gs of maize seedling leaves compared to the control (**Figures 2a–c**). The LHAal, SHA, and CHA treatments significantly reduced the Ci of the leaves (**Figure 2d**). The LHAac, MHA, SHA, and CHA treatments significantly increased the SPAD value of the leaves (**Figure 2e**).

### 3.3 Biomass and nutrient content of maize seedlings under different HA treatments

**Figure 3:**
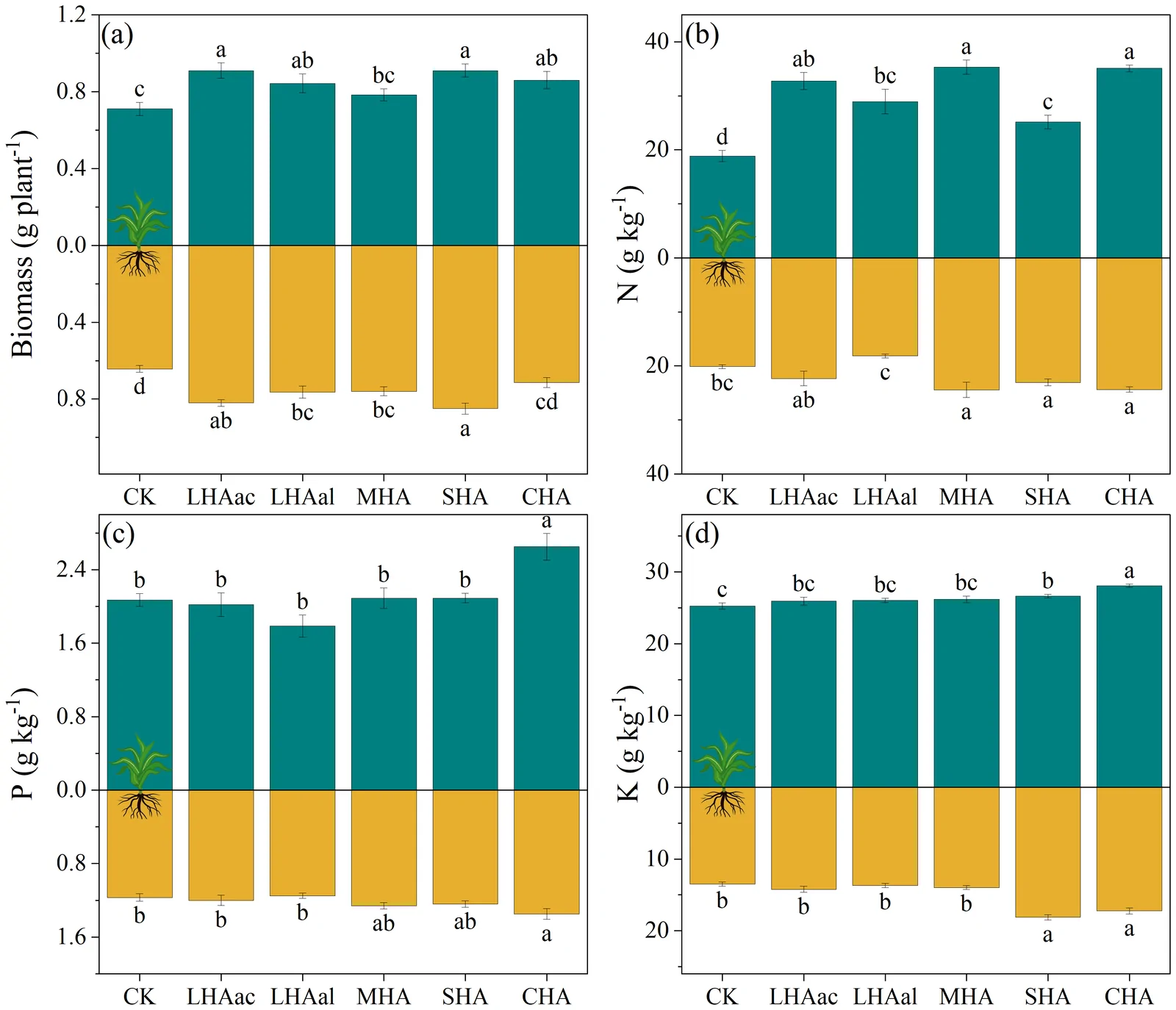
Biomass and nutrient content of maize seedlings under different HA treatments. **(a)** Shoot and root biomass; **(b)** Shoot and root total nitrogen content; **(c)** Shoot and root total phosphorus content; **(d)** Shoot and root total potassium content. Different lowercase letters represent significant differences between treatments.

Compared with the control, all HA treatments increased the shoot and root biomass of maize seedlings (**Figure 3a**). Specifically, the LHAac, LHAal, SHA, and CHA treatments significantly increased shoot biomass by 28.17%, 18.78%, 26.76%, and 21.13%, respectively, compared to the control. The LHAac, LHAal, MHA, and SHA treatments significantly increased root biomass by 15.49%, 7.51%, 7.04%, and 19.72%, respectively. All HA treatments also significantly increased total nitrogen content in shoots of maize seedling shoots, and the MHA, SHA, and CHA treatments significantly increased the total nitrogen content in roots as well (**Figure 3b**). The SHA treatment significantly increased the total phosphorus content in both shoots and roots of the maize seedlings (**Figure 3c**). The SHA and CHA treatments significantly increased the total potassium content in both shoots and roots of the seedlings (**Figure 3d**).

### 3.4 Factors influencing shoot and root biomass of maize seedlings

Maize root biomass was significantly correlated with SPAD, Pn, Gs, Tr, shoot N content, and total root length. Shoot biomass was significantly correlated with Pn, Gs, Tr, shoot N, shoot K, root K, total root length, and root vigor. Moreover, root N, P, and K contents were all positively and significantly correlated with shoot N, P, and K contents, indicating a coordinated improvement in the nutritional status of both roots and shoots.

**Figure 4:**
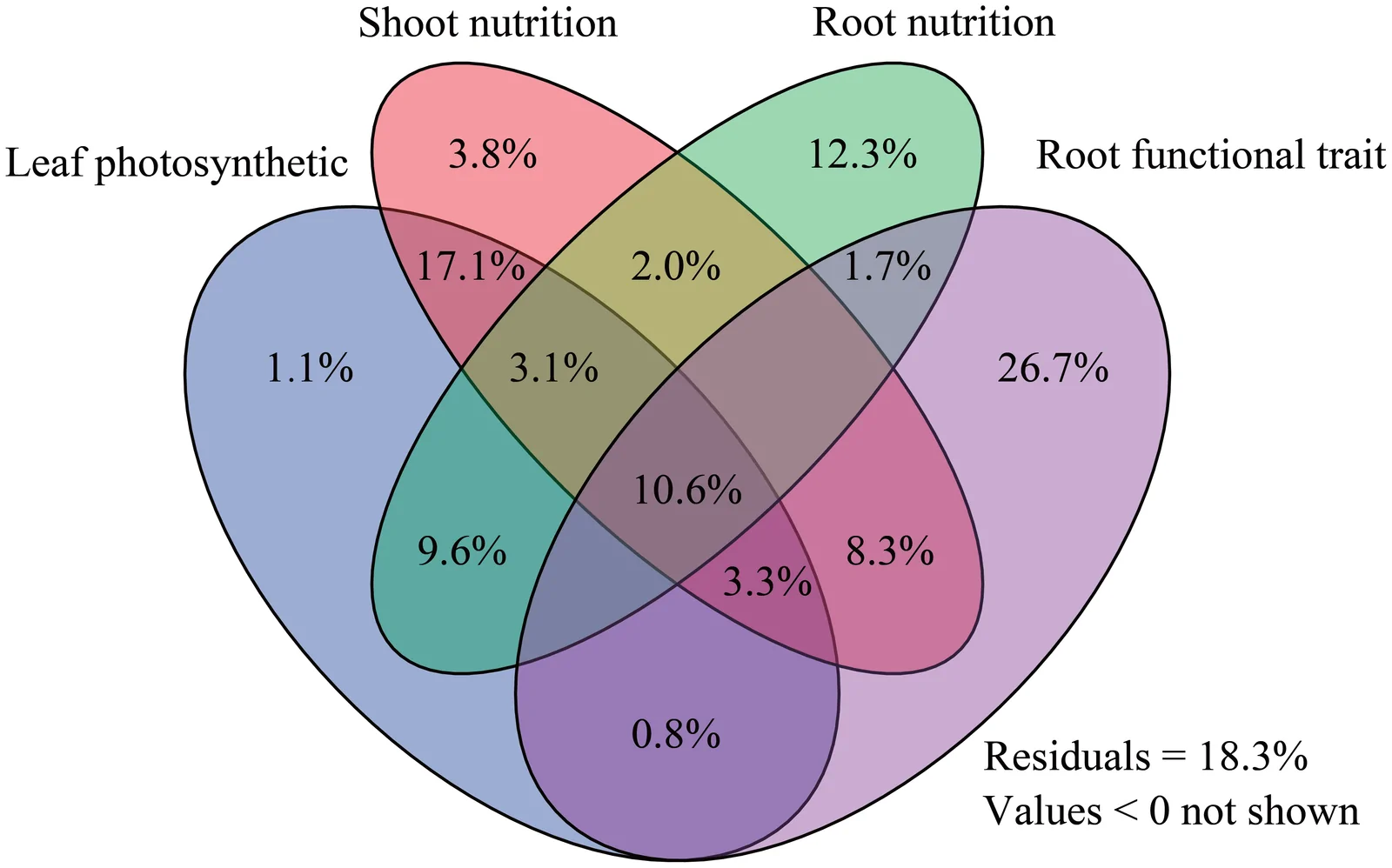
Variation partitioning analysis (VPA) of maize seedling biomass in relation to different categories of growth parameters. Maize seedling biomass is partitioned into shoot biomass and root biomass. Leaf photosynthetic parameters include SPAD, Pn, Gs, and Ci. Shoot nutritional parameters include shoot N, P, and K content. Root nutritional parameters include root N, P, and K content. Root functional traits include total root length, specific root length, and root vigor.

To compare the contributions of leaf photosynthesis, shoot nutrition, root nutrition, and root functional traits to variations in maize seedling biomass, a variance partitioning analysis was performed (**Figure 4**). To eliminate collinearity among parameters, we selected representative parameters from four categories: SPAD, Pn, Gs, and Ci for leaf photosynthetic parameters; shoot N, P, and K contents for shoot nutrition; root N, P, and K contents for root nutrition; and total root length, specific root length, and root vigor for root functional traits. The results showed that root functional traits alone explained the largest portion of biomass variation (26.7%). Root nutrient status alone explained 12.3% of the variation. Leaf photosynthetic parameters and shoot nutrient status each had smaller individual contributions. However, there were notable interactive effects among these factors. For example, the combined effect of leaf photosynthetic capacity and shoot nutrient status explained about 17.1% of the variation. Leaf photosynthesis combined with root nutrient status explained 9.6%. Shoot nutrient status combined with root functional traits explained 10.6%, and leaf photosynthesis combined with root functional traits explained 8.3%. These overlapping contributions indicate that improvements in maize seedling growth under HA treatments result from multiple factors acting synergistically. Overall, HA-induced shifts in root development, photosynthetic performance, and nutrient status jointly correlated with elevated seedling biomass, and root functional traits exhibited the strongest independent explanatory power for biomass variation.

## 4 Discussion

### 4.1 Effects of HA on maize seedling root functional traits

Compared to the control, HA treatments increased seedling total root length by approximately 13%–39%, indicating that all types of HA significantly promoted maize seedling root growth. Studies have shown that the root-promoting effect of HA may be related to its hormone-like activity and stimulation of root ion pumps. Low concentrations of HA can mimic auxin activity, inducing plants to produce more lateral roots and root branches (Canellas and Olivares, 2014; Rathor et al., 2023). The action of HA is analogous to auxin in stimulating lateral root formation in maize, which is consistent with our observation that HA significantly increased total root length (Souza et al., 2022). In addition, HA can promote the synthesis and activity of plasma membrane H^+^-ATPase in root cells, thereby promote root cell elongation and growth; this process directly leads to increased root length and root biomass (Canellas et al., 2002; Souza et al., 2022). Some studies have also reported that HA application can induce the expression of high-affinity phosphate transporter genes in plants, activating the plant’s own nutrient uptake pathways and improving the efficiency of nutrient utilization by the roots (Yuan et al., 2023, Yuan et al., 2024). Consistent with the measured data of this study, HAs from different sources had varying effects on root morphology. The MHA treatment produced seedlings with the greatest total root length and specific root length, and it significantly decreased average root diameter, indicating that MHA promoted the development of fine roots (i.e., an increased proportion of finer roots). In contrast, the SHA treatment resulted in the largest average root diameter and a significantly lower specific root length, reflecting a relatively thicker root system under that treatment. Although the roots were thicker with the SHA treatment, the root tissue density was significantly higher, indicating a more compact root biomass (more dry matter per root volume).

The above differential root responses are speculated to be closely related to the distinct composition and properties of HAs from different sources, according to previous studies. The source of HA determines its molecular structure and functional group content, which influence its biological activity (de Melo et al., 2016; Nardi et al., 2021). Generally, MHA (such as those extracted from weathered coal) is rich in aromatic rings. Following alkaline extraction and oxidation, they may yield more low-molecular-weight humic substances that contain numerous carboxyl and phenolic hydroxyl groups and have high bioactivity (Gong et al., 2020; Yang et al., 2021; Jing et al., 2022). High-molecular-weight humic fractions can stimulate plasma membrane H^+^-ATPase activity, whereas low-molecular-weight fractions can more easily penetrate into plant cells to act as signaling molecules (Zandonadi et al., 2025). Combined with our experimental results, MHA likely contained small-molecule humic/fulvic acid components that effectively stimulated root growth. On the other hand, SHA comes from materials rich in nutrients and contains a relatively high proportion of fulvic acid components; it has a smaller molecular size and a high content of functional groups, enabling it to provide nutrients while directly stimulating root metabolism (Jindo et al., 2012; Cristina et al., 2020). The types and amounts of functional groups differ greatly between HAs; for instance, HAs rich in carboxyl groups can more strongly promote plant nitrogen uptake (Ampong et al., 2022; Jing et al., 2022). Sludge-derived HAs typically have higher nitrogen content and abundant polar functional groups, which help enhance plant nitrogen metabolism and increase root vigor (Jindo et al., 2012). In summary, MHA tended to markedly promote root elongation and fine root formation, SHA was more effective at increasing root thickness and metabolic activity, whereas LHA and CHA HAs had more intermediate, balanced effects.

### 4.2 Effects of HA on maize seedling photosynthetic characteristics

Our results demonstrate that all HA treatments increased the net photosynthetic rate (Pn), stomatal conductance (Gs), and transpiration rate (Tr) of maize seedling leaves (**Figures 2a–c**), indicating an overall enhancement of photosynthetic capacity and transpiration in the plants. After HA application, the relative chlorophyll content (SPAD value) of leaves was also generally higher than in the control; in particular, the LHAac, MHA, SHA, and CHA treatments significantly increased SPAD (**Figure 2e**), suggesting that HA treatments increased leaf chlorophyll content. This is consistent with other findings that HA can improve plant uptake of macro- and micronutrients and stimulate the synthesis of growth-promoting hormones, thereby raising leaf chlorophyll concentration and the activity of photosynthetic enzymes (Chen et al., 2022; Jing et al., 2022). Adequate nutrient supply, particularly sufficient nitrogen, directly increases leaf chlorophyll content, thereby enhancing photosynthetic efficiency. This study’s data confirmed that HA treatments increased the total nitrogen content in the shoots (**Figure 3b**), which provides a basis for chlorophyll synthesis. As reported in previous studies, HAs can chelate and transport micronutrients (such as Fe and Zn) into plants, improving the availability of these elements (Shah et al., 2018). Iron in particular is essential for chlorophyll synthesis, and HA can increase iron availability, indirectly promote chlorophyll formation, and thus increase SPAD values (Turan et al., 2022). Additionally, studies have indicated that HA can increase levels of endogenous hormones in plants and enhance the activity of photosynthesis-related enzymes, leading to an increase in the photosynthetic rate (Chen et al., 2021). Slight differences were observed among the HA treatments in terms of leaf gas exchange parameters. In the LHAal, SHA, and CHA treatments, the intercellular CO_2_ concentration (Ci) of maize leaves was significantly lower than that of the control (**Figure 2d**), whereas Ci in the LHAac and MHA treatments did not differ significantly from that in the control. This difference may reflect the balance between photosynthetic rate and stomatal opening under different treatments. Generally, a decrease in Ci indicates that CO_2_ is being rapidly assimilated by the mesophyll cells due to enhanced photosynthesis, resulting in a relative decline in intercellular CO_2_ concentration (Flexas et al., 2007). In the LHAal, SHA, and CHA treatment groups, Pn was greatly increased along with higher Gs, indicating that leaves were taking in and assimilating CO_2_ more efficiently, which manifested as a lower Ci. This suggests that HA treatments not only promoted stomatal opening (providing more CO_2_) but also enhanced the internal capacity of leaves to assimilate CO_2_. In contrast, although the MHA treatment significantly increased Pn and Gs, it did not lead to a significant reduction in Ci, possibly implying that the improvement in photosynthesis under MHA was driven mainly by improved stomatal conductance, and the mesophyll’s CO_2_ assimilation capacity had not become a limiting factor. Despite some differences in the mechanisms of photosynthetic enhancement among the treatments, all HA treatments resulted in higher Pn. This can be attributed to improved overall plant nutritional and water status: HA-treated plants had more developed root systems with enhanced water and nutrient uptake ability, providing leaves with sufficient water and nutrients to maintain higher stomatal conductance and transpiration rates, and thus sustaining a higher level of photosynthesis (Rathor et al., 2024; Zandonadi et al., 2025). In summary, HAs increased the photosynthetic performance of maize seedlings through multiple pathways, such as boosting leaf chlorophyll content and stomatal conductance, thereby laying a foundation for greater biomass accumulation.

### 4.3 Effects of HA on maize seedling biomass and nutrient content

The biomass promotion effect observed in this study is consistent with previous research conclusions. All types of HA increased the shoot and root dry weight of maize seedlings compared to the control. The promotive effect of humic substances on plant growth has been widely documented: it has been reported that HA extracted from lignite can increase the biomass of gerbera and rapeseed; other studies have shown that HA extracted from organic waste significantly increased the dry weight of crop roots and shoots (Eyheraguibel et al., 2008; Nikbakht et al., 2008; Kłeczek and Anielak, 2021; Zhylina et al., 2025). Consistent with the above literature, our study verified that HA application significantly increased maize seedling shoots and root biomass, indicating a coordinated enhancement of both belowground and aboveground growth. Consistent with previous reports, HA application increased both root and shoot dry weight, which proved the obvious synergistic effect between root and shoot growth (Chen and Aviad, 1990; Chen et al., 2022). On one hand, HA stimulates root development, thereby improving the plant’s ability to absorb water and nutrients (Zandonadi et al., 2025). On the other hand, HA itself contains nutrients and can improve soil nutrient dynamics by increasing fertilizer use efficiency and reducing nutrient losses (Nardi et al., 2021). The numerous anionic functional groups in HA can form chelates with cationic nutrient ions, preventing nutrients from being fixed in the soil or leaching away, and allowing them to be released slowly for plant uptake (de Melo et al., 2016; Mindari et al., 2024). Moreover, HA can stimulate roots to secrete organic acids and enzymes that help solubilize otherwise insoluble nutrients; for instance, HA has been shown to induce the expression of phosphate transporter genes in plants, thereby enhancing phosphorus uptake (Jing et al., 2021; Zhang et al., 2024b). Our results demonstrated that HA treatments significantly improved the nutrient status of maize seedlings, facilitating greater nutrient accumulation in the plants. All HA treatments notably increased the total nitrogen content in the shoots, and the MHA, SHA, and CHA treatments also significantly increased the total nitrogen in the roots (**Figure 3b**). The SHA treatment significantly elevated the total phosphorus content in both shoots and roots (**Figure 3c**), and the SHA and CHA treatments significantly raised the total potassium content in both shoots and roots (**Figure 3d**). These results indicate that HAs enhanced the uptake of N, P, and K by maize seedlings. Sludge-derived HA (SHA) is rich in organic N and P compounds that decompose to release nutrients slowly; it also contains abundant carboxyl and phenolic functional groups that can chelate soil cations such as potassium and calcium, thereby reducing nutrient leaching. This may explain why the SHA treatment led to significant increases in seedling N, P, and K contents (Stevenson, 1994; Lamar et al., 2024). Mineral-source HA (MHA) itself has low nutrient content, but it can improve root architecture and thereby indirectly enhance nutrient uptake. Biological HA (LHA) primarily contributes organic matter and some potassium; its direct nutrient supply is limited, but by improving soil conditions and stimulating root growth, it can also help improve nutrient acquisition by plants. In summary, when applying HAs in agricultural production, their source and composition should be considered alongside conventional nutrient inputs in combination with conventional nutrient inputs, in order to achieve the dual objectives of improving nutrient use efficiency and promoting plant growth.

### 4.4 Analysis of factors influencing maize seedling biomass accumulation

**Figure 5:**
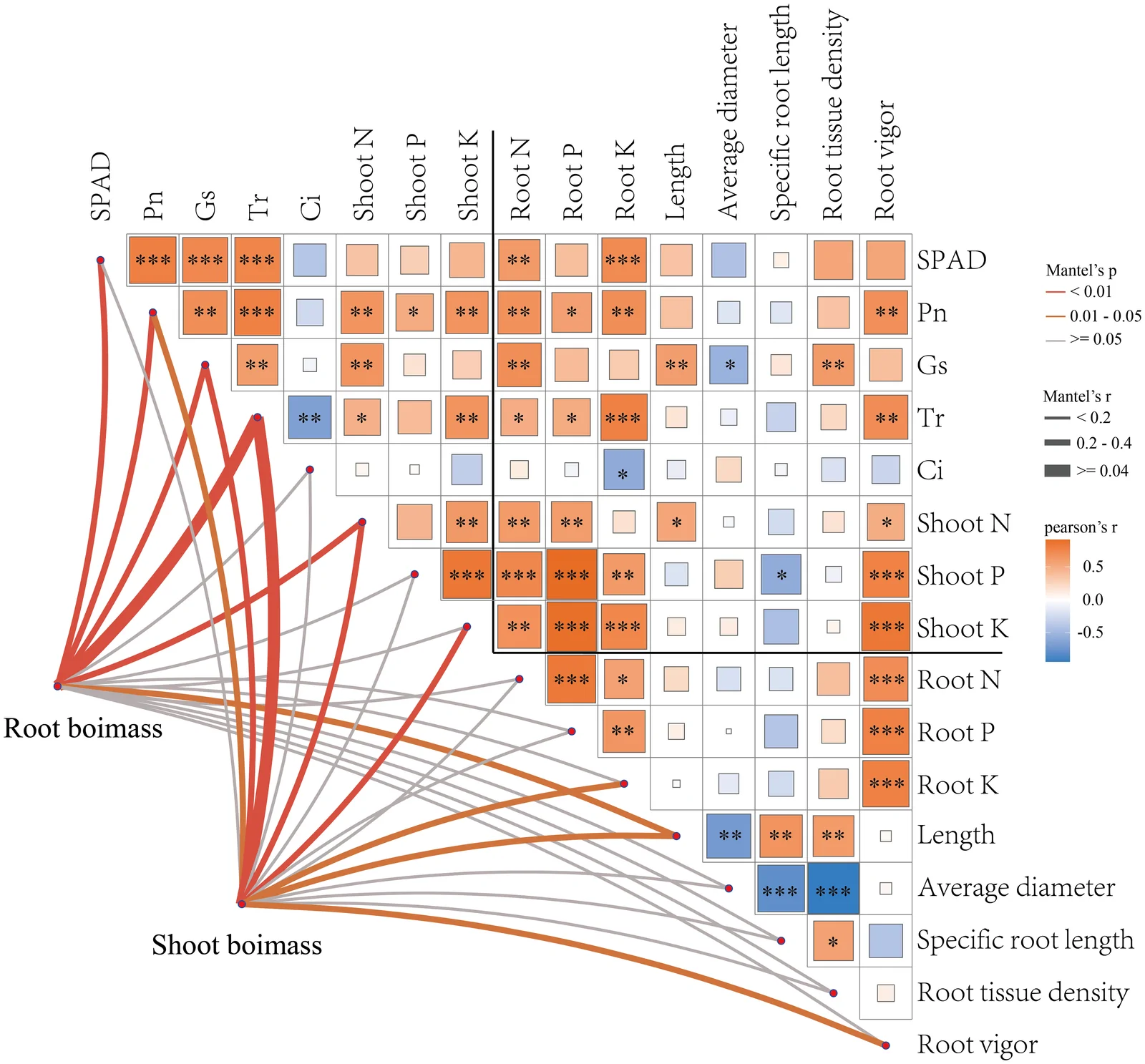
Shows the Mantel test results for correlations between maize seedling biomass and various parameters related to nutrients, photosynthesis, and root traits. *P<0.05, **P<0.01, and ***P<0.001.

In this study, Mantel tests and VPA were used to explore the relationships between various growth indices and maize seedling biomass, and to quantify their relative contributions. Mantel test results (**Figure 5**) showed that root biomass was significantly positively correlated with leaf SPAD, Pn, Gs, Tr, shoot N content, and total root length. Shoot biomass was significantly positively correlated with Pn, Gs, Tr, shoot N content, shoot K content, root K content, total root length, and root vigor. Additionally, root N, P, and K contents were all significantly positively correlated with shoot N, P, and K contents, indicating a synchronized improvement in the plant’s nutritional status above- and below-ground (Chen and Aviad, 1990; Chen et al., 2022).

Variance partitioning analysis (**Figure 4**) further demonstrated the relative importance of different factors for biomass accumulation. Among the four categories of factors considered (leaf photosynthetic parameters, shoot nutrient status, root nutrient status, and root functional traits), root functional traits had the largest independent contribution, explaining about 26.7% of the variation in seedling biomass. Root nutrient status alone explained roughly 12.3% of the variation. The independent contributions of leaf photosynthetic parameters and shoot nutrient status were smaller. However, these factors also showed considerable interactive effects on biomass. For example, the combination of leaf photosynthetic capacity and shoot nutrient status explained an additional 17.1% of the variation; leaf photosynthesis combined with root nutrient status explained 9.6%. Shoot nutrient status combined with root functional traits explained 10.6%, and leaf photosynthesis combined with root functional traits explained 8.3%. These interactive effects indicate that under HA treatments, improvements in plant growth result from multiple factors acting in concert. In summary, HA-mediated increases in maize seedling biomass were associated with coordinated changes in root functional morphology, whole-plant nutrient status, and leaf photosynthetic capacity, and according to variance partitioning analysis, root functional traits made the largest independent contribution to the observed biomass variation.

## 5 Conclusions

All tested sources of HA were found to significantly promote the growth of maize seedlings in gray desert soil. Compared with the control, each HA treatment generally improved maize seedling root development (increasing total root length, producing finer roots, and increasing root tissue density and root vigor) and leaf photosynthetic capacity (increasing Pn, Gs, and Tr, raising SPAD, and lowering Ci). These changes led to significantly higher shoot and root biomass of the maize seedlings, along with increased plant nitrogen, phosphorus, and potassium contents. Among the different HAs, the mineral-source HA (MHA) had the most pronounced effect on promoting fine root growth, whereas the sludge-derived HA (SHA) markedly increased root thickness and root tissue density. All HA treatments effectively enhanced leaf chlorophyll content and net photosynthetic rate, thereby improving the overall nutritional status of the plants. Mantel tests and VPA results indicated that HA-induced alterations in root functional traits, photosynthetic performance, and nutrient uptake were all closely correlated with elevated maize seedling biomass, and root functional traits accounted for the largest share of biomass variation among the measured plant indicators. These findings provide an important reference for the targeted selection of HA products according to actual production needs in agricultural production. However, the results of this study are based on pot experiments, and further field-scale validation is needed to clarify the application effects of different HAs under actual field conditions.

## Data Availability

The datasets presented in this article are not readily available because the dataset associated with this article is subject to the following restrictions: Partial public availability Aggregated numerical data underlying the core findings (including root morphological traits, leaf gas exchange parameters, plant biomass, and tissue nutrient concentrations) are fully presented in the article and accessible to all readers. The complete raw experimental datasets (raw root scanning imagery, raw output files from the LI-6800 system, full batch records of soil and plant chemical analysis) are institutional assets of the Xinjiang Academy of Agricultural Sciences, generated under an ongoing funded research project. These full raw datasets are not publicly available at present to safeguard the project’s intellectual property and enable follow-on research work. Controlled access for academic use Eligible researchers may request access to the full raw datasets by contacting the corresponding authors. Requests must specify the intended research purpose and scope of data use. Access will be approved exclusively for non-commercial academic research, subject to a data use agreement and written approval from the project’s host institution. Prohibition on redistribution and commercial use Approved users may not redistribute, sublicense, or republish any portion of the raw dataset in any form, nor use it for commercial purposes, without prior written consent from the corresponding authors and the Xinjiang Academy of Agricultural Sciences. These restrictions do not compromise the validity or reproducibility of the core conclusions reported in this work. Requests to access the datasets should be directed to Ding Zhaolong, dingzhaolong2411@163.com.

## References

[B1] Akimbekov N. Qiao X. Digel I. Abdieva G. Ualieva P. Zhubanova A. (2020). The effect of leonardite-derived amendments on soil microbiome structure and potato yield. Agriculture 10, 147. doi: 10.3390/agriculture10050147 30654563

[B2] Ali S. Kumari A. Dhyani V. Chaturvedi S. (2025). Humic acid and phosphate solubilizing bacteria led phosphorous bioavailability for enhancing photosynthetic efficiency and productivity of direct-seeded rice. Soil Environ. 44, 86–91. doi: 10.25252/SE/2025/253726

[B3] Ampong K. Thilakaranthna M. S. Gorim L. Y. (2022). Understanding the role of humic acids on crop performance and soil health. Front. Agron. 4, 848621. doi: 10.3389/fagro.2022.848621

[B4] Cai S. Zhang Y. Hu A. Liu M. Wu H. Wang D. . (2023). Dissolved organic matter transformation mechanisms and process optimization of wastewater sludge hydrothermal humification treatment for producing plant biostimulants. Water Res. 235, 119910. doi: 10.1016/j.watres.2023.119910 37001233

[B5] Canellas L. P. Olivares F. L. (2014). Physiological responses to humic substances as plant growth promoter. Chem. Biol. Technol. Agric. 1, 3. doi: 10.1186/2196-5641-1-3 38164791

[B6] Canellas L. P. Olivares F. L. Okorokova-Façanha A. L. Façanha A. R. (2002). Humic acids isolated from earthworm compost enhance root elongation, lateral root emergence, and plasma membrane H+-ATPase activity in maize roots. Plant Physiol. 130, 1951–1957. doi: 10.1104/pp.007088 12481077 PMC166705

[B7] Chen Q. Qu Z. Li Z. Zhang Z. Ma G. Liu Z. . (2021). Coated diammonium phosphate combined with humic acid improves soil phosphorus availability and photosynthesis and the yield of maize. Front. Plant Sci. 12. doi: 10.3389/fpls.2021.759929 34975942 PMC8716685

[B8] Chen Q. Qu Z. Ma G. Wang W. Dai J. Zhang M. . (2022). Humic acid modulates growth, photosynthesis, hormone and osmolytes system of maize under drought conditions. Agric. Water Manage. 263, 107447. doi: 10.1016/j.agwat.2021.107447 38826717

[B9] Chen Y. Aviad T. (1990). “ Effects of humic substances on plant growth,” in Humic Substances in Soil and Crop Sciences: Selected Readings (Madison, Wisconsin: American Society of Agronomy, Crop Science Society of America, and Soil Science Society of America), 161–186. doi: 10.2136/1990.humicsubstances.c7

[B10] Cristina G. Camelin E. Ottone C. Fraterrigo Garofalo S. Jorquera L. Castro M. . (2020). Recovery of humic acids from anaerobic sewage sludge: Extraction, characterization and encapsulation in alginate beads. Int. J. Biol. Macromol. 164, 277–285. doi: 10.1016/j.ijbiomac.2020.07.097 32673726

[B11] da Silva R. Olivares F. Peres L. Novotny E. Canellas L. (2025). Humic substances promote the activity of enzymes related to plant resistance. Agriculture 15, 1688. doi: 10.3390/agriculture15151688 30654563

[B12] de Melo B. A. G. Motta F. L. Santana M. H. A. (2016). Humic acids: Structural properties and multiple functionalities for novel technological developments. Mater. Sci. Eng. C 62, 967–974. doi: 10.1016/j.msec.2015.12.001 26952503

[B13] Eyheraguibel B. Silvestre J. Morard P. (2008). Effects of humic substances derived from organic waste enhancement on the growth and mineral nutrition of maize. Bioresour. Technol. 99, 4206–4212. doi: 10.1016/j.biortech.2007.08.082 17962015

[B14] FAO (2011). Save and grow: a policymaker's guide to the sustainable intensification of smallholder crop production (Rome: Food and Agriculture Organisation of the United Nations).

[B15] Flexas J. Diaz-Espejo A. Galmés J. Kaldenhoff R. Medrano H. Ribas-Carbo M. (2007). Rapid variations of mesophyll conductance in response to changes in CO2 concentration around leaves. Plant Cell Environ. 30, 1284–1298. doi: 10.1111/j.1365-3040.2007.01700.x 17727418

[B16] Gong G. Xu L. Zhang Y. Liu W. Wang M. Zhao Y. . (2020). Extraction of fulvic acid from lignite and characterization of its functional groups. ACS Omega 5, 27953–27961. doi: 10.1021/acsomega.0c03388 33163778 PMC7643152

[B17] Guo H. Wu Z. Li J. Lu Z. Luan S. Hu S. . (2025). A nine-year study: continuous application of biochar achieves efficient potassium supply by modifying soil clay mineral composition and its potassium adsorption sites. J. Soils Sediments 25, 1829–1845. doi: 10.1007/s11368-025-04035-5 30311153

[B18] Hao Z. Zhang S. Shao Y. Pan Z. Meng T. Liu T. . (2025). Preparation and inoculation of Bacillus spp. and Sinorhizobium meliloti strains immobilized on biochar-humic acid improve potted soybean traits and soil parameters. Environ. Technol. Innovation 38, 104210. doi: 10.1016/j.eti.2025.104210 38826717

[B19] Höfle D. Sperber S. Marzban N. Antonietti M. Hoffmann T. Wicaksono W. . (2024). Artificial humic acid diminishes the effect of drought on the soil microbiome. J. Sustain. Agric. Environ. 3 (4), e70034. doi: 10.1002/sae2.70034 41531421

[B20] Jarosz R. Kowalska J. B. Gondek K. Bejger R. Mielnik L. Lahori A. H. . (2025). The effect of new zeolite composites from fly ashes mixed with leonardite and lignite in enhancing soil organic matter. Agriculture 15, 786. doi: 10.3390/agriculture15070786 30654563

[B21] Jindo K. Martim S. A. Navarro E. C. Pérez-Alfocea F. Hernandez T. Garcia C. . (2012). Root growth promotion by humic acids from composted and non-composted urban organic wastes. Plant Soil 353, 209–220. doi: 10.1007/s11104-011-1024-3 30311153

[B22] Jing J. Y. Yuan L. Zhang S. Q. Li Y. T. Zhao B. Q. (2021). Effects and mechanism of humic acid in humic acid enhanced phosphate fertilizer on fertilizer-phosphorus migration. Sci. Agric. Sin. 54, 5032–5042. doi: 10.3864/j.issn.0578-1752.2021.23.009

[B23] Jing J. Zhang S. Yuan L. Li Y. Chen C. Zhao B. (2022). Humic acid modified by being incorporated into phosphate fertilizer increases its potency in stimulating maize growth and nutrient absorption. Front. Plant Sci. 13, 885156. doi: 10.3389/fpls.2022.885156 35665178 PMC9161291

[B24] Juan Q. P. Chapin L. J. Martins E. M. Jones M. L. (2024). A biostimulant containing humic and fulvic acids promotes growth and health of tomato ‘Bush beefsteak’ plants. Horticulturae 10, 671. doi: 10.3390/horticulturae10070671 30654563

[B25] Kłeczek A. Anielak A. M. (2021). Humic substances and significance of their application–a review. Tech. Trans. 118 (1), e2021012. doi: 10.37705/TechTrans/e2021012 35584415

[B26] Kumar Sootahar M. Zeng X. Wang Y. Su S. Soothar P. Bai L. . (2020). The short-term effects of mineral-and plant-derived fulvic acids on some selected soil properties: improvement in the growth, yield, and mineral nutritional status of wheat (Triticum aestivum L.) under soils of contrasting textures. Plants 9, 205. doi: 10.3390/plants9020205 32041329 PMC7076438

[B27] Lal R. (2015). Restoring soil quality to mitigate soil degradation. Sustainability 7, 5875–5895. doi: 10.3390/su7055875 30654563

[B28] Lamar R. T. Gralian J. Hockaday W. C. Jerzykiewicz M. Monda H. (2024). Investigation into the role of carboxylic acid and phenolic hydroxyl groups in the plant biostimulant activity of a humic acid purified from an oxidized sub-bituminous coal. Front. Plant Sci. 15, 1328006. doi: 10.3389/fpls.2024.1328006 38751833 PMC11095639

[B29] Liang T. Elmaadawy K. Liu B. Hu J. Hou H. Yang J. (2021). Anaerobic fermentation of waste activated sludge for volatile fatty acid production: Recent updates of pretreatment methods and the potential effect of humic and nutrients substances. Process. Saf. Environ. Prot. 145, 321–339. doi: 10.1016/j.psep.2020.08.010 38826717

[B30] Ma Y. Cheng X. Zhang Y. (2024). The impact of humic acid fertilizers on crop yield and nitrogen use efficiency: a meta-analysis. Agronomy 14 (12), 2763. doi: 10.3390/agronomy14122763

[B31] MacCarthy P. (2001). The principles of humic substances. Soil Sci. 166, 738–751. doi: 10.1097/00010694-200111000-00003 42348292

[B32] Mahoney K. J. McCreary C. Depuydt D. Gillard C. L. (2017). Fulvic and humic acid fertilizers are ineffective in dry bean. Can. J. Plant Sci. 97, 202–205. doi: 10.1139/cjps-2016-0143 34819996

[B33] Mindari W. Chakim M. G. Widjajani B. W. Sasongko P. E. Aditya H. F. Pazi A. M. M. . (2024). The optimization of biosilica and humic acid to increase soil nutrient availability and nutrient uptake in rice plant in sandy soil. Caraka Tani 40, 18–33. doi: 10.20961/carakatani.v40i1.89018

[B34] Mondher R. Oleiwi M. (2025). “ Effect of adding agricultural sulphur and spraying with humic acid on the availability of some nutrients and soil sustainability,” in IOP Conference Series: Earth and Environmental Science, vol. 1487. , 012181. doi: 10.1088/1755-1315/1487/1/012181

[B35] Nabi F. Sarfaraz A. Kama R. Kanwal R. Li H. (2025). Structure-based function of humic acid in abiotic stress alleviation in plants: A review. Plants 14, 1916. doi: 10.3390/plants14131916 40647925 PMC12251879

[B36] Nardi S. Schiavon M. Francioso O. (2021). Chemical structure and biological activity of humic substances define their role as plant growth promoters. Molecules 26, 2256. doi: 10.3390/molecules26082256 33924700 PMC8070081

[B37] Nikbakht A. Kafi M. Babalar M. Xia Y. P. Luo A. Etemadi N. (2008). Effect of humic acid on plant growth, nutrient uptake, and postharvest life of gerbera. J. Plant Nutr. 31, 2155–2167. doi: 10.1080/01904160802462819 37339054

[B38] Rathor P. Gorim L. Y. Thilakarathna M. S. (2023). Plant physiological and molecular responses triggered by humic based biostimulants-a way forward to sustainable agriculture. Plant Soil 492, 31–60. doi: 10.1007/s11104-023-06156-7 30311153

[B39] Rathor P. Upadhyay P. Ullah A. Gorim L. Y. Thilakarathna M. S. (2024). Humic acid improves wheat growth by modulating auxin and cytokinin biosynthesis pathways. AoB Plants 16, plae018. doi: 10.1093/aobpla/plae018 38601216 PMC11005776

[B40] Shah Z. H. Rehman H. M. Akhtar T. Alsamadany H. Hamooh B. T. Mujtaba T. . (2018). Humic substances: Determining potential molecular regulatory processes in plants. Front. Plant Sci. 9, 263. doi: 10.3389/fpls.2018.00263 29593751 PMC5861677

[B41] Sheteiwy M. S. Ahmed M. Alyafei M. Sulieman S. Ali B. Jośko I. . (2025). Sugar metabolism and metabolite profiling of maize grains are largely improved by the combination of arbuscular mycorrhizal fungi and humic acid. Ind. Crops Prod. 234, 121546. doi: 10.1016/j.indcrop.2025.121546 38826717

[B42] Souza A. Olivares F. Peres L. Piccolo A. Canellas L. (2022). Plant hormone crosstalk mediated by humic acids. Chem. Biol. Technol. Agric. 9 (1), 29. doi: 10.1186/s40538-022-00295-2 38164791

[B43] Stevenson F. J. (1994). Humus Chemistry: Genesis, Composition, Reactions (New York, NY: John Wiley & Sons).

[B44] Tavares O. C. H. Santos L. A. Filho D. F. Ferreira L. M. Garcia A. C. Castro T. A. V. T. . (2021). Response surface modeling of humic acid stimulation of the rice (Oryza sativa L.) root system. Arch. Agron. Soil Sci. 67, 1046–1059. doi: 10.1080/03650340.2020.1775199 37339054

[B45] Turan M. Ekinci M. Kul R. Kocaman A. Argin S. Zhirkova A. M. . (2022). Foliar applications of humic substances together with Fe/nano Fe to increase the iron content and growth parameters of spinach (Spinacia Oleracea L.). Agronomy 12, 2044. doi: 10.3390/agronomy12092044 30654563

[B46] Wang F. Sun H. Ren X. Liu Y. Zhu H. Zhang P. . (2017). Effects of humic acid and heavy metals on the sorption of polar and apolar organic pollutants onto biochars. Environ. pollut. 231, 229–236. doi: 10.1016/j.envpol.2017.08.023 28802992

[B47] Yang F. Tang C. Antonietti M. (2021). Natural and artificial humic substances to manage minerals, ions, water, and soil microorganisms. Chem. Soc Rev. 50, 6221–6239. doi: 10.1039/D0CS01363C 34027951

[B48] Yuan Y. Tang C. Jin Y. Cheng K. Yang F. (2023). Contribution of exogenous humic substances to phosphorus availability in soil-plant ecosystem: A review. Crit. Rev. Environ. Sci. Technol. 53, 1085–1102. doi: 10.1080/10643389.2022.2120317 37339054

[B49] Yuan Y. Yang F. Liu Z. Cheng K. (2024). Artificial humic acid improves P availability via regulating P-cycling microbial communities for crop growth. Plant Soil 512 (1), 1–18. doi: 10.1007/s11104-024-07100-z 30311153

[B50] Zandonadi D. B. Monda H. Gralian J. James A. Lamar R. T. Santos M. P. (2025). Humic acids as drivers of plant growth: regulating root development and photobiology through redox modulation. Chem. Biol. Technol. Agric. 12, 71. doi: 10.1186/s40538-025-00789-9 38164791

[B51] Zhang F. Chen X. Vitousek P. (2013). Chinese agriculture: An experiment for the world. Nature 497, 33–35. doi: 10.1038/497033a 23636381

[B52] Zhang Z. Ma Y. Tian Y. Liu P. Zhang M. Liu Z. . (2024b). Co-application of coated phosphate fertilizer and humic acid for wheat production and soil nutrient transport. Agronomy 14, 1621. doi: 10.3390/agronomy14081621 30654563

[B53] Zhang J. Meng Q. Yang Z. Zhang Q. Yan M. Hou X. . (2024a). Humic acid promotes the growth of switchgrass under salt stress by improving photosynthetic function. Agronomy 14, 1079. doi: 10.3390/agronomy14051079 30654563

[B54] Zhao W. Xiao J. Wang S. Gai X. Chen G. (2025). Bone biochar and humic acid improved soil quality and promoted Olea europaea growth in coastal saline soil by enhancing the stoichiometric homeostasis of nutrient elements. Biochar 7, 70. doi: 10.1007/s42773-025-00461-3 30311153

[B55] Zhou Q. Wu S. Xing L. Wang Z. Zhang X. Zhang F. . (2025). Molecular mechanisms of biostimulants in promoting tomato seedling growth: Linking chemical structure to physiologic function. J. Agric. Food. Chem. 73, 7632–7644. doi: 10.1021/acs.jafc.4c12283 40119812

[B56] Zhylina M. Karnozhytskyi P. Miroshnichenko D. Konohrai V. Sterna V. Ozolins J. (2025). The effect of growth stimulants based on humic acids from Ukrainian lignite and biochar from agricultural residues on the growth and development of lettuce (Lactuca sativa). Agron. Res. 23, 571–584. doi: 10.15159/ar.25.013

